# Radical mediastinal lipectomy for tamponade-like cardiac physiology

**DOI:** 10.1186/s13019-023-02421-z

**Published:** 2023-11-22

**Authors:** Mohsyn Imran Malik, James Changhyun Jae, Osama Sedky Shehata Sefein, Raffael Pereira Cezar Zamper, A. Dave Nagpal

**Affiliations:** 1 Division of Cardiac Surgery, London Health Science Centre/Western University, Suite B6-104, 339 Windermere Rd, London, ON N6A 5A5 Canada; 2 Department of Anesthesia and Perioperative Medicine, London Health Sciences Centre/Western University, London, ON Canada; 3 Critical Care Western, London Health Sciences Centre/Western University, London, ON Canada

**Keywords:** Tamponade, Pericardial fat, Mediastinal fat, Reoperation, Echocardiography

## Abstract

**Background:**

Re-opening the chest is an unwanted and potentially morbid complication after open heart surgery, most commonly required for refractory bleeding or tamponade. In this report, we present a unique case of a postoperative coronary artery bypass patient, demonstrating clinical features of cardiac tamponade of the right atrium and ventricle with inconclusive findings on imaging.

**Case presentation:**

A 62 year-old male presented to hospital with exertional angina and a coronary angiogram found severe three-vessel coronary artery disease with preserved left ventricular function. He underwent an uncomplicated triple coronary artery bypass surgery. Over the following hours in the cardiac intensive care unit, the patient had a climbing serum lactate level and increasing vasopressor requirements. On investigations, there was evidence of compression of the right heart. The patient was taken back to the operating room where very little clot or bleeding was identified, rather there was significant amounts of mediastinal fat surrounding the heart which was subsequently resected with wide margins. The patient had complete resolution of their symptoms and an uncomplicated postoperative course thereafter.

**Conclusions:**

To our knowledge, this case is the first reported occurrence of cardiac constriction from excessive mediastinal fat after open heart surgery. Identifying patients at high-risk for excessive pericardial fat, as well as considering alternative modalities of imaging appear to be the main stay in diagnosis at this point. Current treatment is a mediastinal lipectomy with wide margins, avoiding injury to surrounding structures such as the phrenic nerve and innominate vein. Future study might consider the value of prophylactic mediastinal lipectomy at time of surgery, and methods to improve detection with current and future imaging modalities.

**Supplementary Information:**

The online version contains supplementary material available at 10.1186/s13019-023-02421-z.

## Background

Re-opening the chest is an unwanted and potentially morbid complication after open heart surgery, most commonly required for refractory bleeding or tamponade. Re-exploration for bleeding is associated with increased length of hospital stay, higher risk of sternal wound infection, renal impairment, and postoperative arrythmias [1]. As such, measures are taken pre-, intra-, and post-operatively to prevent the need for re-exploration.

In this report, we present a unique case of a postoperative coronary artery bypass patient, demonstrating clinical features of cardiac tamponade of the right atrium and ventricle with inconclusive findings on imaging. On re-exploration, no significant clots or bleeding were discovered, but rather a large amount of mediastinal and pericardial fat was found to be compressing the right heart. The patient underwent radical resection of the fat, with subsequent improvement in hemodynamics and an uncomplicated postoperative course thereafter.

## Case presentation

A 62 year-old male presented to hospital with exertional angina and past medical history of hypertension, dyslipidemia, diabetes, obstructive sleep apnea, allergic rhinitis, umbilical herniorrhaphy, and abdominal obesity with a body-mass index of 35. He also had known coronary artery disease with a myocardial infarction in 2007 requiring percutaneous intervention to the right coronary artery. He was initially trialed on medical management for his exertional symptoms, however worsening of the angina prompted a repeat coronary angiogram, which demonstrated severe three-vessel coronary artery disease with preserved left ventricular function.

As such, the patient was consented for coronary artery bypass grafting (CABG), and subsequently underwent a triple CABG with anastomosis of the left internal thoracic artery to the left anterior descending artery, an in-situ skeletonized right internal thoracic artery to a large obtuse marginal artery, and a left endo-radial artery to the posterior descending artery. Large amounts of pericardial fat were noted at the time of opening of the pericardium. Total cardiopulmonary bypass time was 58 min and total cross-clamp time was 44 min. No blood products were required, and good graft flows were confirmed with a Medistim flowmetry device [Medistem ASA, Norway]. Postoperative transesophageal echocardiogram (TEE) demonstrated normal biventricular function. The patient was brought to the Cardiac Surgery Recovery Unit in normal sinus rhythm, without any inotrope or vasopressor support.

**Fig. 1:**
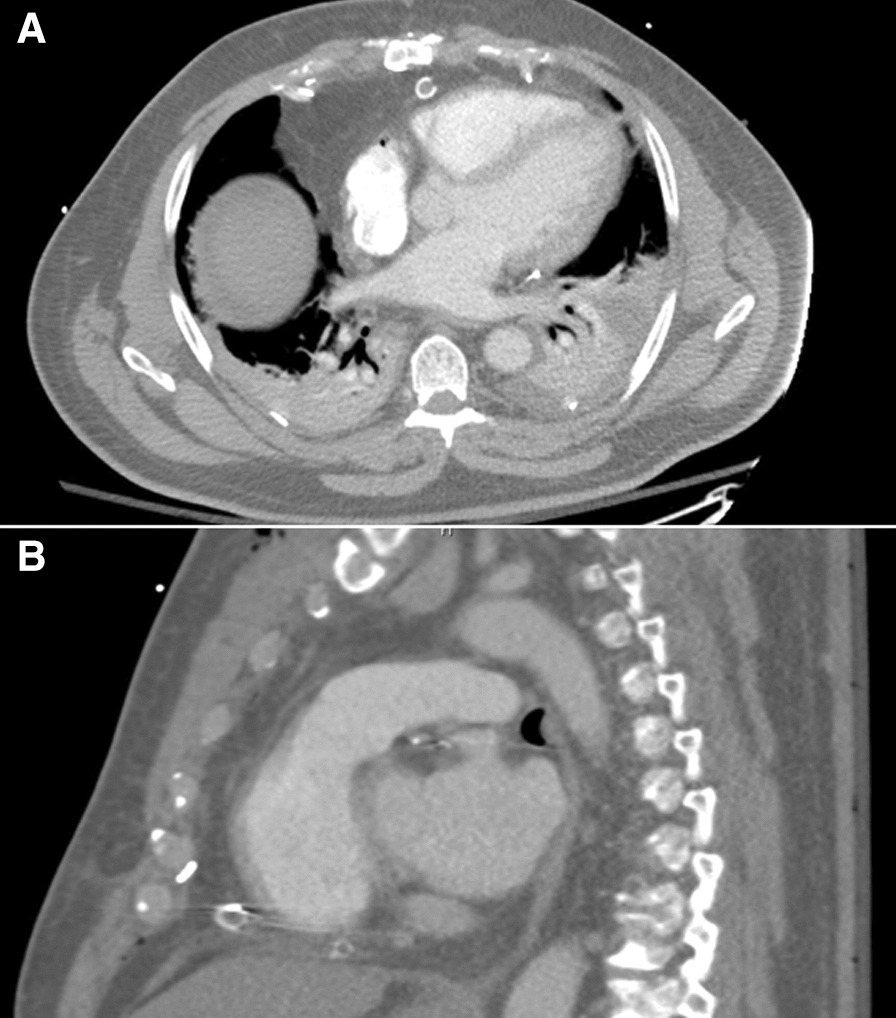
**A** Axial and **B** sagittal computed tomography images demonstrating large amounts of soft tissue around the right ventricle and right atrium

Over the next few hours in the recovery unit, his serum lactate began to rise. The patient was initially responsive to fluid resuscitation, however, the lactate continued to trend upwards and by the next morning, was as high as 11.3 mmol/L. There was minimal drainage from the mediastinal chest tubes; approximately 300 ml of serosanguinous fluid over 12 h. Three electrocardiograms were performed over the course of the day, all of which were negative for any new S-T changes. A TEE demonstrated a structure compressing the right ventricle (RV) and right atrium (RA), though ventricular function was preserved with no new regional wall motion abnormalities. (Additional file 1A)) A CT Chest was performed to delineate the structure along the right heart and significant mediastinal and paracardiac fat was identified, though without evidence of compression (Fig. 1). Despite a positive fluid balance of 5.5 L, vasopressors continued to increase, with a peak norepinephrine of 16 mcg/minute and vasopressin of 2.4 units/h. Given the hemodynamic instability and inability to wean mechanical ventilation, the decision was made to take the patient back to the operating room for re-exploration, approximately 24 h after his initial operation.

**Fig. 2:**
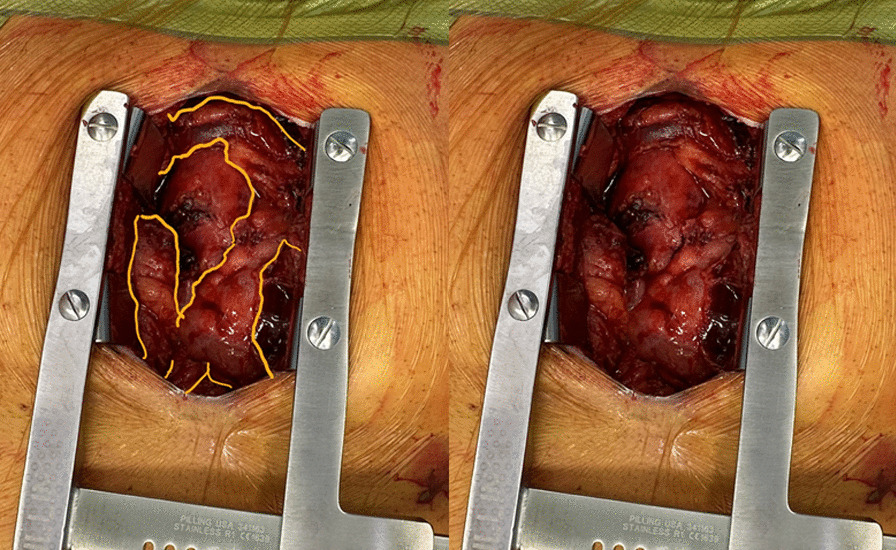
**A** Intraoperative photo demonstrating large amounts of mediastinal fat engulfing the heart. **B** Outlining of the pericardial fat relative to the small area of cardiac structures visible on median sternotomy

**Fig. 3:**
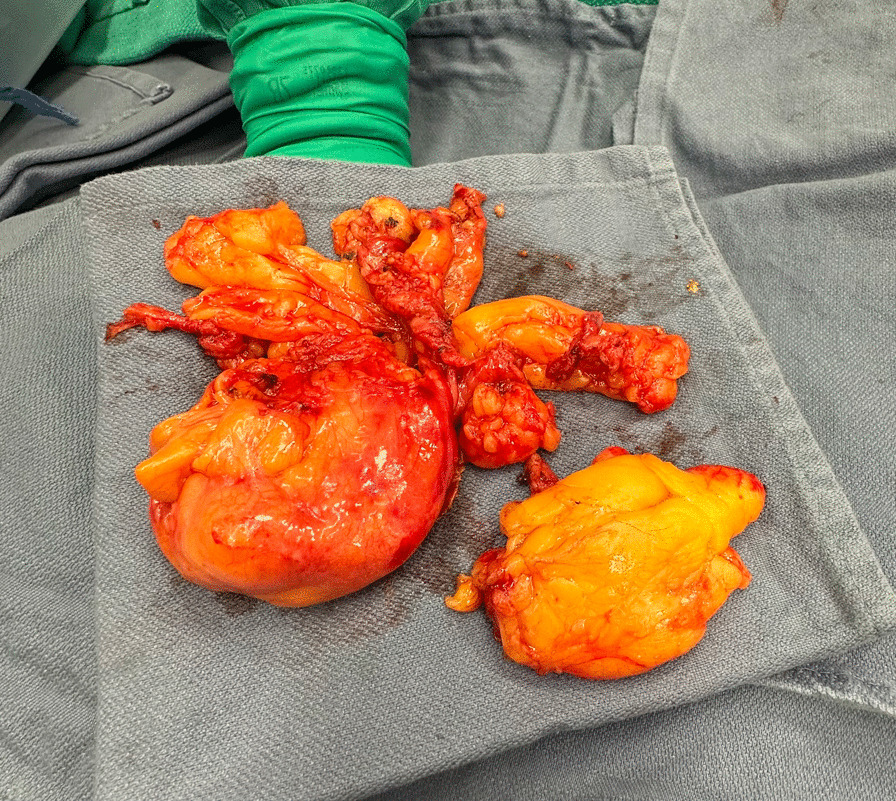
Total pericardial and mediastinal lipomatous tissue removed after re-opening of the median sternotomy

In the operating room, the previous sternotomy incision was reopened and sternal wires removed. Minimal thrombus was encountered in the mediastinum. However, a large amount of (now edematous) fat was identified encircling the heart. As such a radical anterior lipectomy was performed with electrocautery, including the entire thymus, bilateral pleural fat pads, and mediastinal fat superiorly around the innominate vein and inferiorly, down to the pericardio-diaphragmatic junction (Fig. 2). A total of 250 g of fat was removed (Fig. 3). Hemostasis was ensured, and new mediastinal chest tubes were inserted before closing the chest.

Upon completion, the patient continued to be in normal sinus rhythm, however, now required half the vasopressors needed prior to the case. Additionally, the transesophageal echocardiogram revealed improved RA and RV filling. (Additional file 1B) The patient was brought back to the Cardiac Surgery Recovery Unit, where he was extubated and completely weaned from vasoactive medications shortly thereafter. He continued to progress well, and was discharged home on postoperative day 5 with no outstanding concerns.

## Discussion and conclusions

To our knowledge, this case is the first reported occurrence of cardiac tamponade from excessive mediastinal fat after open heart surgery. There have been few reported cases of this similar pathology, though under difference circumstances. A report from 2004 describes a patient misinterpreted to have a pericardial effusion based on transthoracic echocardiography, and instead found to have pericardial lipotamous hypertrophy on cardiac MRI. They underwent an elective pericardiotomy and large excisions were made anterior to the phrenic nerves to allow decompression. The patient’s symptoms were relieved and follow up cardiac MRI showed normal function and increased filling of the right ventricle [2].

In 2006, a patient on chronic steroids therapy was persistently hypotensive, and echocardiogram was suggestive of a moderate pericardial effusion with tamponade features. However, a cardiac MRI confirmed the compressive force to be secondary to pericardial fat, requiring elective surgical management [3]. From a 2009 report, a post-myocardial infarction patient was found on urgent transthoracic echocardiogram to have a moderate sized pericardial effusion along the right ventricular free wall, with an adherent mass thought to be a thrombus. It was initially thought this may be secondary to post-myocardial infarction pericarditis, or worse, a free-wall rupture. On emergent surgical exploration, there was no evidence of fluid or thrombus, however a large amount of epicardial fat which was removed, with resolution of the tamponade physiology [4].

Thus, excessive pericardial fat leading to constriction and re-exploration appears to be a novel complication in postoperative cardiac surgery patients. Based on the existing literature, transthoracic and transesophageal echocardiogram remain the gold-standard for diagnosing cardiac tamponade, though cardiac MRI is an ideal adjunct for differentiating tissue from thrombus in stable patients [2, 3]. However, undergoing a cardiac MRI can be challenging in the acute postoperative period. In this case, CT Chest provided additional information which informed the decision to go back to the operating room, and thus may be a suitable alternative in the perioperative period.

With the pericardotomy, this excess fat was released, sliding laterally to the heart chambers leading to compression which may have be worsened over the course of the day by swelling and hematoma of the pericardial and thymic fat. This is supported by the final pathology which identified the specimen as “fragments of mature adipose tissue with areas of acute congestion and fibrin deposition”. Thus, high index of suspicion for constrictive pericardial fat or “fat tamponade” should be maintained in postcardiotomy patients with tamponade physiology who are known to have significant pericardial fat on opening of the chest, who have minimal mediastinal chest tube output and/or have risk factors for excessive pericardial fat, such as chronic steroid use, intraabdominal fat, insulin resistance, and metabolic syndrome, in general [5].

While this appears to be a relatively uncommon cause of re-exploration in post cardiac surgery patients, we believe that with the increasing prevalence of metabolic syndrome in our patients, it is an important differential to consider in selected cases. Identifying patients at high risk for excessive pericardial fat, as well as considering alternative modalities of imaging appear to be the main stay in diagnosis at this point. Current treatment is a mediastinal lipectomy with wide margins, avoiding injury to surrounding structures such as the phrenic nerve, and innominate vein. Future study would be helpful to determine the value in prophylactic mediastinal lipectomies at time of surgery, and to further delineate methods to improve detection with current and future imaging modalities.

### Supplementary Information

**Additional file 1**. (A) Pre- and (B) post- resection transesophageal echocardiogram videos demonstrating preserved biventricular systolic function, with changes in right atrial and ventricle filling during diastole.

## Data Availability

Not applicable.
